# Just Google It: Young Children’s Preferences for Touchscreens versus Books in Hypothetical Learning Tasks

**DOI:** 10.3389/fpsyg.2016.01431

**Published:** 2016-09-22

**Authors:** Sierra Eisen, Angeline S. Lillard

**Affiliations:** Department of Psychology, University of Virginia, Charlottesville, VAUSA

**Keywords:** learning, touchscreen devices, educational tools, books, children’s education

## Abstract

Children today regularly interact with touchscreen devices (Rideout, 2013) and thousands of “educational” mobile applications are marketed to them (Shuler, 2012). Understanding children’s own ideas about optimal learning has important implications for education, which is being transformed by electronic mobile devices, yet we know little about how children think about such devices, including what children think touchscreens are useful for. Based on a prior result that children prefer a book over a touchscreen for learning about dogs, the present study explored how children view touchscreens versus books for learning an array of different types of information. Seventy children ages 3–6 were presented with six different topics (cooking, today’s weather, trees, vacuums, Virginia, and yesterday’s football game) and chose whether a book or a touchscreen device would be best to use to learn about each topic. Some of this information was time-sensitive, like the current weather; we predicted that children would prefer a touchscreen for time-sensitive information. In addition, each child’s parent was surveyed about the child’s use of books and touchscreens for educational purposes, both at home and in school. Results indicated that younger children had no preference between books and touchscreen devices across learning tasks. However, 6-year-olds were significantly more likely to choose the touchscreen for several topics. Surprisingly, 6-year-olds chose a touchscreen device to learn about time-sensitive weather conditions, but not yesterday’s football. Children’s choices were not associated with their use of books and touchscreens at home and school.

## Introduction

Children’s use of touchscreen devices has grown tremendously in the last decade. In a 2013 nationwide survey by Common Sense Media, 72% of children below the age of eight used a mobile device – almost twice as many as in 2011 (Rideout, 2011, 2013). Although considerable attention has been paid to the “digital divide” between the technology access of lower- and higher-income families (e.g., Attewell, 2001; Wartella et al., 2013), recent research suggests that mobile use in low-income families is robust (Kabali et al., 2015). Kabali et al. (2015) surveyed an urban, low-income, minority community and found that 96.6% of children under the age of four had used mobile devices. Even by the age of two, over 75% of low-income children used mobile devices on a daily basis, more than four times the 17% rate reported by Common Sense Media two years prior (Rideout, 2013).

Children use mobile devices to watch videos, to play games, to read, to communicate with others, and increasingly, to learn. Educational applications abound in the touchscreen app marketplace and the majority are marketed toward children and teenagers (Shuler, 2012). Yet as recent reviews have highlighted, a severe lack of regulation hinders the ability of parents to choose educational apps wisely (Guernsey et al., 2012; Hirsh-Pasek et al., 2015). Parents hold varying attitudes about the educational benefits of media use. For example, 37% of parents claim mobile devices have a positive effect on their child’s reading skills, while 21% claim a negative effect, and 40% claim a neutral effect (Wartella et al., 2013). The majority of parents of children under the age of eight are likely to use a book instead of a technological tool to educate their children, although this varies with age: 64% of parents with 6–8-year-old children say they would direct their child to a computer in order to learn (Wartella et al., 2013). Although 67% of parents claim books are very important sources of learning, only 44% claim interactive digital media are valuable for learning (Rideout, 2014). Parental attitudes toward media predict children’s actual media use (Lauricella et al., 2015) and the extent to which parents view media as having educational value predicts their children’s use of educational media tools (Cingel and Krcmar, 2013). Parents’ own use also predicts their children’s use, although parental attitudes toward media affect child use even when parents themselves are infrequent users (Lauricella et al., 2015). For instance, parents who have positive rather than negative attitudes toward tablets have children who spend more time with tablets, even if the parents are only low or medium tablet users. Thus, children’s media use can be affected by both parental use and parental attitude, as well as by factors of age and availability (Lauricella et al., 2015; Rideout, 2011, 2013).

Increasingly, researchers are evaluating children’s ability to learn from touchscreen devices and educational apps. In contrast to the literature on learning from television, which has consistently found that children fail to transfer information from screens to the real world (Barr and Hayne, 1999; Anderson and Pempek, 2005; Krcmar et al., 2007; Roseberry et al., 2009; DeLoache et al., 2010), studies examining learning from touchscreens have presented mixed results. Recent studies have shown that young children learned equally well from touchscreens and physical objects in a problem-solving task (Huber et al., 2015) and that nightly engagement with a math app increased children’s math achievement, particularly for children whose parents were anxious about math (Berkowitz et al., 2015). Yet other studies indicate that young children have difficulty transferring between 2D touchscreens and 3D objects (Zack et al., 2009, 2013; Moser et al., 2015), presumably due to the challenge of extending new information beyond the specific context in which it was learned, though this may be most pronounced in infants (Barr, 2013). In a recent comparison of different learning tools, children learned geography better from a physical puzzle than an app version of the puzzle in an initial interaction with the tool (Eisen and Lillard, 2016). After children brought home either the puzzle or the app for 1 week, the degree of advantage was reduced and children who used the puzzle learned only marginally more than those who used the app; however, children used the app for twice as long as the puzzle over the week, suggesting that learning from the puzzle was more efficient. Further research on children’s learning from touchscreen devices is greatly needed, especially considering how rapidly touchscreens have been integrated into classrooms across the country (Richtel, 2011).

One unexplored aspect of the topic is whether children view touchscreen devices as tools for learning. Children begin to discuss learning and teaching during the preschool years (Bartsch et al., 2003) and by the age of six they recognize that learning requires not just a desire to learn but attention to the task (Sobel et al., 2007). Yet when asked about new pieces of knowledge, preschoolers often claim they have always known the information (Taylor et al., 1994; Esbensen et al., 1997). Furthermore, 3-year-olds struggle to remember sources of learning, particularly after a delay (Gopnik and Graf, 1988), whereas 4- and 5-year-olds can remember sources but not when something was learned (Tang and Bartsch, 2012). By the age of four, children can generate details about how their own learning takes place (Bemis et al., 2011, 2013) but their ability to conceptualize and accurately describe learning develops well into the elementary school years (Sobel and Letourneau, 2015). In an open-ended interview, Sobel and Letourneau (2015) asked 4–10-year-old children about their concept of learning. Older children understood learning as process-based and gave answers that reflected learning strategies. In contrast, 4- and 5-year-old children often struggled to answer the questions, although approximately 40% described learning as a process by referring to either a source (such as a teacher) or a strategy (such as practice). Putting these findings together, it appears that by 4 years old, children’s concept of learning in sufficiently developed to sensibly answer a question regarding the best source of learning.

To learn from a source, one must also evaluate that source as trustworthy and informative. This is just as true for technological sources as it is for social sources. Building off of the large literature on children’s trust in human informants (e.g., Koenig et al., 2004; Jaswal and Neely, 2006; Birch et al., 2008), Danovitch and Alzahabi (2013) asked preschoolers to evaluate the accuracy of computer informants. Children as young as three showed selective trust in an accurate computer over an inaccurate computer. When asked to explain the errors of an inaccurate computer, 4- and 5-year-olds claimed the errors reflected the computer’s lack of knowledge, not human error. This study indicates that young children understand that despite holding a wealth of information, computers are not infallible. Further research suggests that children initially trust human informants over technological informants, but by the age of five, children endorse technological over human informants (Noles et al., 2015). Adults favor technological informants as well, in both their endorsements and information seeking.

Relatedly, as children learn to read, they prioritize printed information over oral information (Einav et al., 2013; Eyden et al., 2013; Robinson et al., 2013). Early readers use printed labels to correct their own guesses and believe printed labels over oral labels (Robinson et al., 2013), even when the printed labels conflict with children’s own impressions (Eyden et al., 2013). In contrast, pre-readers do not show the same affinity for print, although along with early readers, they may reject information that is printed but seems incorrect.

Do children recognize that touchscreen devices can be valuable sources of information? Eisen and Lillard (2015) showed preschoolers ages four to six images of various objects, including a book, iPad, and iPhone, and asked if the objects could be used to learn. Surprisingly, only 53.5% of children said that an iPad could be used for learning, and just 34.9% said an iPhone could be. In comparison, 81.4% of children said a book could be used for learning. Children were also asked to choose which object would be best for them to use for learning about dogs in a hypothetical scenario and the majority of children chose the book. These results indicate that children may privilege books over touchscreens in the context of learning, which is surprising given how attracted children can be to electronic devices. However, it is possible that when presented with an actual learning task using real objects, children would choose an electronic device over a book.

In the present study, children were offered a variety of topics to learn about and asked to choose between two potential learning tools: a book and a touchscreen. Eisen and Lillard (2015) found differences between smartphones and tablets in children’s assessment of learning capacity, so we included both types of touchscreen to further explore these differences. Since Apple devices have dominated the touchscreen market for the last five years (King, 2015) and have been used in prior studies (Berkowitz et al., 2015; Huber et al., 2015), we used an iPad and an iPhone and referred to them by these names. The learning topics presented to children were chosen to cover a wide range of subjects that could be learned about in a variety of ways, including by using a book or touchscreen. We were also interested in whether children recognize the advantage of using a touchscreen to procure certain types of information, particularly variable, time-sensitive information. For example, if one wanted to learn about weather in the general sense, a book could be just as helpful as a touchscreen device. However, if one wanted to learn about *today’s* weather, a touchscreen would be the more appropriate tool. To explore this, we included two learning topics for which it would be best to use a touchscreen, to assess whether children treat timely information differently. Thus our study included two types of learning topics: general and time-sensitive. Although we found no prior research on children’s comprehension of time-sensitive information, we believe that because touchscreens are frequently used to learn this type of information, children may recognize this particular benefit of touchscreens. Parents were surveyed about their children’s use of books and touchscreens to learn in different settings. Based on prior research (Eisen and Lillard, 2015), we predicted that children would prefer books as a learning tool for our general topics. We further expected that children would recognize that time-sensitive information is best gained from using a touchscreen device. Lastly, we predicted that children who use touchscreens frequently for educational purposes would favor the touchscreen device in our task.

## Materials and Methods

### Participants

Seventy children participated, including eighteen 3-year-olds (*M* = 41.05 months, *SD* = 3.12, range = 37.1 – 45.8; 8 female), seventeen 4-year-olds (*M* = 55.36 months, *SD* = 3.51, range = 49.8 – 59.8; 7 female), eighteen 5-year-olds (*M* = 66.42 months, *SD* = 3.89, range = 60.4 –71.1; 9 female), and seventeen 6-year-olds (*M* = 78.72 months, *SD* = 4.11, range = 72 – 83.5; 9 female). Specific data on children’s ethnicity was not collected but children were predominantly white and middle class, reflecting the families who volunteer for research in the community. Children were recruited from a local children’s museum and from a database of families willing to bring their children to the laboratory for research. Parents provided written informed consent for their child’s participation, approved by the host institution’s research ethics committee. Children provided verbal assent to the experimenter before entering the testing room. Parents and children were debriefed after the study. An additional five children were tested but excluded from analysis due to inattention (3) or inability to complete the experiment (2).

### Materials

The materials consisted of six books, each measuring 9 by 6.5 cm in size and 20 pages in length, as well as a black iPad mini and a white iPhone 6. Each book had a distinct cover to represent each of the six learning topics, which were: cooking, today’s weather, trees, vacuum cleaners, Virginia, and yesterday’s football game. Each cover displayed an image to represent the topic. For example, the cooking cover showed an image of a chef holding a plate of pasta and the Virginia cover showed an image of the state of Virginia. The touchscreen devices displayed PDF versions of these covers. To maintain consistent object positions, the books and touchscreen devices were presented to the child on a blue plastic tray measuring 45 by 30.5 cm. A female doll named “Sarah” was also presented to children for each trial.

### Procedure

Participants were first introduced to Sarah the doll, which sat at the far end of the table and faced the child. The experimenter explained that Sarah wanted to learn about different topics and that she had different tools she could use to learn, but that she needed the child’s help to make her choices. Underneath the table and out of sight of the participant, the experimenter placed the first book and a touchscreen device onto the tray, then lifted the tray onto the center of the table in front of the child. Whether the touchscreen device was an iPad or an iPhone was counterbalanced, as was the position of each object on the tray. For half of the participants, topics were displayed in a fixed order (trees, cooking, weather, Virginia, vacuum cleaners, and football) and for the other half of participants, the order was reversed. The six topics were chosen to cover a wide range of information that would likely be familiar to children but not so common that they would have prior experience learning about the topics using books or touchscreens. After the experimenter placed the tray with the learning tools on the table, she explained that Sarah wanted to learn about a particular topic (e.g., trees) and that Sarah had a book about that topic and an iPad (or an iPhone) with an app about that topic. A doll was chosen as “the learner” so that children would not take into account their own or the experimenter’s prior knowledge about the topics. The experimenter pointed to each object as it was introduced and the order of introduction was counterbalanced. The experimenter than asked the participant to choose which tool Sarah should use to learn about the topic and explain why Sarah should use the tool. This process was repeated for all six learning topics.

Explanations of children’s learning choices were coded into seven discrete categories: *preference*, in which children mention preference or desire (e.g., “She wants to”), *learning*, in which children explicitly reference learning (e.g., “I use the iPhone to learn”), *comparison*, in which children contrast the two tools (e.g., “A book has more words about it”), *action*, in which children describe a physical action that can be done with the tool (e.g., “It can scroll”), *topic-specific*, in which children directly reference the topic at hand (e.g., “It has planting”), *object-specific*, in which children directly reference an aspect of the tool (“Phones can do anything”), and *no response*, including responses of “I don’t know” or “I’m not sure.” A research assistant, blind to the purpose of the experiment, coded the entire dataset of explanations. A second blind research assistant coded 25% of the dataset. Interrater reliability was high (kappa = 0.88) and discrepancies were resolved through discussion with the first author.

While children were being tested, parents filled out a questionnaire about their child’s use of books and touchscreens to learn at home and in school. Parents were asked whether their child primarily uses touchscreens for educational, entertainment, or other purposes. Parents were also asked about the child’s personal experience or observations of others’ learning about the study’s specific topics from a book or a touchscreen, to account for the role of experience in children’s responses. Finally, parents were questioned about their personal beliefs of the educational merits of books and touchscreens. Appendix A includes the full parent questionnaire.

## Results

Overall, children in our sample frequently used books, with 87.1% reading or being read to daily and the remaining 12.9% reading several times a week (see **Table 1**). Touchscreen use was more variable, with 45.7% of children using them daily, 41.4% using them weekly, and 12.9% using them less than once a week. This frequency of touchscreen use falls between the levels reported by other studies in recent years (Kabali et al., 2015; Rideout, 2013). Fisher’s exact test revealed significant age differences in level of touchscreen use between 5-year-olds and all other ages, *p* = 0.04, with a much higher frequency (77.8%) of 5-year-olds shown to be daily users. No age differences were found for children’s frequency of reading books, as the vast majority of children were daily readers.

**Table 1:** Frequency of use of learning tools.

		Low (Less than once a week)	Medium (Weekly)	High (Daily)
3 years	Book	0	16.6	83.4
	Touchscreen	22.2	38.9	38.9
4 years	Book	0	5.9	94.1
	Touchscreen	17.6	53	29.4
5 years	Book	0	11.1	88.9
	Touchscreen	5.6	16.6	77.8
6 years	Book	0	17.6	82.4
	Touchscreen	5.9	58.9	35.2
Total	Book	0	12.9	87.1
	Touchscreen	12.8	41.4	45.7

*Frequencies are shown as percentages. The total includes frequency of use for all ages.*

**FIGURE 1:**
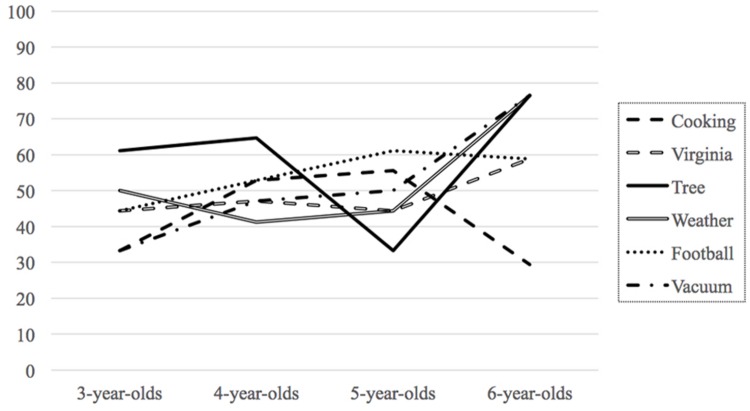
**Percentage of touchscreen choices for each topic by age group.** The percentage of book choices is not shown but is the inverse of this graph.

Preliminary Chi-Squared analyses revealed no effects of touchscreen type (iPad or iPhone), order, or gender on children’s learning choices, so these variables were collapsed in subsequent analyses. The percentages of touchscreen choices for each task at each age are shown in **Figure 1**. First, responses to each learning choice question were compared against chance performance (50%) for each age group using Binomial tests. For 3-, 4-, and 5-year-olds, learning choices did not differ from chance and children were equally likely to choose the book or the touchscreen for each learning scenario. For 6-year-olds, the touchscreen was chosen significantly more than chance for the tree question (13 out of 17, or 76%, *p* = 0.049), the weather question, (13 out of 17, or 76%, *p* = 0.049), and the vacuum question, (13 out of 17, or 76%, *p* = 0.049). For the cooking question, 6-year-olds showed some preference for the book, although not significantly more than chance (12 out of 17, or 71%). For the Virginia question and the football question, the choices of 6-year-olds did not differ from chance.

Another approach to the data, rather than look at whether a touchscreen device was used more than chance for each item, is to look at whether children at different ages distinguish among the options; that is, do they choose the touchscreen device more for one type of information than another? Because these analyses were based on categorical data, we performed non-parametric analyses for each age group across all learning choice questions. Cochran’s Q test indicated that responses did not differ among the six questions for the 3-, 4-, or 5-year-olds. However, responses did differ among the six questions for 6-year-olds, *Q*(5) = 13.704, *p* = 0.018. Pairwise comparisons with McNemar’s test revealed that 6-year-olds chose the book over the touchscreen significantly more for the cooking question than the tree question, *p* = 0.039, the weather question, *p* = 0.008, or the vacuum question, *p* = 0.021.

Learning choice explanations were not related to children’s learning tool choices for the topic of trees, weather, Virginia, and football. For the topic of cooking, learning choice explanations were associated with tool choice, χ^2^ (6, *N* = 70) = 13.03, *p* = 0.043. The association was moderately strong, Cramer’s *V* = 0.43. *Post hoc* comparisons using adjusted standardized residuals show that children who chose the book were more likely to give preference explanations and children who chose the touchscreen were more likely to give object-specific explanations. For the topic of vacuum cleaners, learning choice explanations were associated with tool choice, χ^2^ (6, *N* = 70) = 13.87, *p* = 0.031. The association was moderately strong, Cramer’s *V* = 0.45. *Post hoc* comparisons show that children who chose the book were more likely to give action explanations and children who chose the touchscreen were more likely to give topic-specific explanations.

Interestingly, children’s use of and observation of others’ use of devices at home or school bore no relation to their judgments. Using Pearson’s correlations, we found that children who read books less frequently (several times a week, *n* = 9) were no less likely to choose a book as a source of information than children who read books daily (*n* = 61). We also found no relation between children’s tendency to choose the touchscreen in our task and their overall use of touchscreens at home or school. Children who were considered low in their touchscreen use (less than once a week, *n* = 9) were no less likely to choose a touchscreen device to get information than were children who were considered medium (weekly, *n* = 29) or high (daily, *n* = 32) users of touchscreen devices.

Parents were also asked their beliefs about the extent to which their child learns from books and touchscreens. The majority of parents (85.7%) said their child learns a lot from reading books; the other parents (14.3%) all claimed their child learns somewhat from books. Parents showed much greater variability in their assessment of learning from touchscreens. A third of parents (33.8%) claimed their child learns only a little or not at all from touchscreen devices, 45.6% claimed their child somewhat learns from touchscreens, and 20.6% claimed their child learns a lot from touchscreens. The extent to which parents stated that their child learns from books showed a trend toward being related to children’s learning choice of books, *r*(70) = -0.19, *p* = 0.11, but their belief in touchscreens as a learning tool was not related to children’s choice of touchscreens, *r*(68) = 0.03, *p* = 0.81. Children’s primary use of touchscreens did not relate to their likelihood of choosing the touchscreen in the learning tasks or to their parent’s belief about learning from touchscreens.

## Discussion

This study explored how children compare books, which have long been viewed as an educational tool, with the increasingly available and popular touchscreen. We hypothesized that children would show a preference for using books to learn about a variety of topics. There are several reasons for this expectation. First, when Eisen and Lillard (2015) surveyed children about the various functions of different media tools, the majority of children claimed learning as a function of books. Far fewer children said that touchscreen devices could be used for learning. Children also chose the book over other objects, including touchscreens, in a hypothetical learning scenario. Second, parents may differ in their beliefs about the potential information to be gained from either books or touchscreens. This could affect how parents discuss learning with their children and the extent to which they turn to books or touchscreens when their child wishes to learn. Books are the more conventional method of learning and past studies have shown that parents prefer to use them for educational needs (Wartella et al., 2013). Third, although touchscreen devices are increasingly integrated into some classrooms, the traditional book still reigns supreme in these settings. The consistent use of books within schools may send an implicit message of their utility in education.

Contrary to our hypothesis, we found that children did not favor books to learn in our task. Indeed, younger children showed no preference between books and touchscreens for the variety of topics about which we inquired. Only 6-year-olds showed particular preferences, and although they preferred to use a touchscreen for three of the six scenarios, they did not differ from chance in their choices for the other three. Specifically, 6-year-olds chose to use a touchscreen to learn about trees, today’s weather, and vacuum cleaners. However, 6-year-olds also tended to choose the book over the touchscreen to learn about cooking, although not at a level significantly different from chance. For the two time-sensitive topics, only 6-year-olds recognized the utility of the touchscreen for up-to-date information, and they did so only for the question about today’s weather. It seems rather surprising that children would think a book could provide information about yesterday’s football game, but almost half of them did. Although the specific topics were meant to strike a balance between familiarity and novelty, learning about current weather may have been too common an activity and learning about football may have been too unusual, leading children to favor the touchscreen for the former but not the latter. Similarly, learning about Virginia may have been too novel or broad a concept, such that 6-year-olds were unsure which tool would be better and chose equally between them. Around the age of six, children readily produce examples of learning sources but have more difficulty describing the process of learning (Sobel and Letourneau, 2015). The 6-year-olds in our study could be too young to easily conceptualize how to learn about highly unfamiliar topics, such as football or a state. Future research might explore this topic with older children.

Children’s explanations for their tool preferences illuminated only some of their choices. For the topic of cooking, children who chose the book more frequently referenced their own or the doll’s preferences, as in, “I use that one too” and “She likes books better.” In contrast, children who chose the touchscreen for cooking claimed object-specific reasons, such as, “It could show you a video” or “It has an app.” This may reflect how adults make similar decisions about cooking. Despite the utility of touchscreens for finding recipes or displaying cooking tutorials, many people prefer a traditional cookbook. However, we found no correlation between children’s tool choices and their observation of others using books or touchscreens to learn about cooking. For the topic of vacuums, children who chose the book gave explanations related to action, such as, “She can turn the pages” or “You can read it,” whereas children who chose the touchscreen gave topic-specific rationales, such as, “It has a lot about vacuum cleaners” or “You can see which [vacuum] you want.” It is not clear why children’s explanations differed for this topic, but one possibility is that children who chose the book interpreted the question as being about manual learning, and therefore linked to physical action, whereas children who chose the touchscreen interpreted the question as “capable of learning about vacuums” in a more general sense. For all other learning topics, children’s explanations were not related to their choice of tool.

Interestingly, we found no relation between children’s general use of touchscreens and books and their choices in our learning task. This was unexpected, since we predicted that children who frequently used touchscreen devices would be more aware of their potential as learning tools, either through personal experience or due to parental beliefs about the educational merit of touchscreens (Cingel and Krcmar, 2013). Most parents reported regular use of books and expressed the belief that their child learned a great deal from reading or being read to. In contrast, although most parents reported their child’s touchscreen use to be at least weekly, parents varied in their belief that learning takes place during these interactions, with a third of parents reporting minimal learning. As Wartella et al. (2013) determined in their survey of parental attitudes, parents are still on the fence about the instructional value of touchscreens and apps. Although parents’ failure to see touchscreens as educational tools could theoretically impact their children’s conceptualization of these devices as paths to learning, we found no relation between parent beliefs and children’s judgments.

Danovitch and Alzahabi (2013) suggest that older children do trust technological devices as sources of information, sometimes even more than human information sources, and that adults actually prefer a technological informant. For adults, this is largely because we are aware that a touchscreen device, via its connection to the Internet, allows for unlimited information, whereas a person (or a book) is inherently finite in knowledge. Young children may lack this understanding. In fact, it is not until late in elementary school that children begin to comprehend the complexity of the Internet, and late in middle school that adolescents understand its social complexity on an adult level (Yan, 2005, 2006, 2009). Therefore, the younger children in our sample were likely unaware of the advantage the touchscreen held over the book. Yet this does not explain the choices of the 6-year-olds, who favored the touchscreen for half of the learning scenarios. Although children who were frequent touchscreen users were not more likely to choose the touchscreen in our study, they may still have a more developed understanding of the utility of touchscreen devices than their younger counterparts, perhaps due to more years of experience with touchscreens rather than greater frequency of use. Since we did not question parents about their children’s past use of touchscreens, this can only be speculated.

This study had several limitations, the first of which is the restricted age range that was tested. An interesting future direction would be to examine how adults respond to these learning scenarios. It seems likely that adults will privilege the touchscreen device for learning, particularly given its integration into everyday life and the access it provides to infinite information. However, adults may also recognize that information from the Internet is often scattered, shallow, and potentially incorrect, leading them to favor books. This study was further limited by a relatively small sample size, which restricts the extrapolation of our findings. A larger sample size and an expansion of the age range to include older children and adults would enable better generalizability of the results.

Methodologically, this study differed from Eisen and Lillard (2015) in three important ways. First, in our learning scenarios children were asked to choose between two actual objects, rather than several images of objects, which we believe aided the validity of our study. Second, while children in Eisen and Lillard (2015) were asked which general object would be best for learning about a particular subject, we specified that both the book and the touchscreen (via an app) held specific information pertinent to the subject. Lastly, children were asked how a doll should make choices between each object, rather than how they themselves should make choices for their own learning objectives. Although the doll was used so that children would not take their own knowledge about the topics into account, this may have led to the different findings of each study. Perhaps children associate books with their own learning but recognize that others can learn from varied sources. The high level of book use in our sample lends support to this idea, since parents report their children learn more often from books than from touchscreen devices.

Finally, although we aimed for a broad range of learning topics for our experiment, by no means did we cover the wide variety of topics that children may use a book or touchscreen to learn about. Instead, we offered children learning scenarios that were realistic, distinctive, and could plausibly be accomplished via either tool. Future research should explore whether children’s learning choices vary by domain. For example, since 6-year-olds in our study primarily chose the touchscreen to learn about trees, would they also choose the touchscreen to learn about other biological organisms? Children may favor using touchscreens or books for specific topics that were not covered by this study.

As children gain independence and agency through early childhood, they have more control over how they gather information. By examining the choices children make between different tools for learning, we can better understand optimal ways to teach them. In this study, we demonstrated that young children view books and touchscreens as equally viable methods of education. By the age of six, children show more distinct opinions about which tool is better and often judge the touchscreen as superior. As touchscreen devices are increasingly used in educational settings, we should continue to explore children’s understanding of their instructive capabilities.

## Author Contributions

SE and AL designed the study, developed the methodology, and wrote the manuscript. SE collected the data and performed the analyses.

## Conflict of Interest Statement

The authors declare that the research was conducted in the absence of any commercial or financial relationships that could be construed as a potential conflict of interest.
