# Supplementary material for: Just Google It: Young Children’s Preferences for Touchscreens versus Books in Hypothetical Learning Tasks
Source: Front Psychol. 2016 Sep 22;7:1431. doi: 10.3389/fpsyg.2016.01431 (PMC5031770; doi:10.3389/fpsyg.2016.01431)
Supplement: Supplementary file 1 [file Presentation_1.PDF]

Appendix A

Parent Questionnaire

1. Does your child attend school?

2. How often does your child use touchscreen devices like tablets/smartphones at home?

________ Several times a day
________ Once a day
________ Several times a week
________ Once a week
________ Less than once a week
________ Never

3. What does your child primarily use touchscreen devices for? Check one option below.

________ Games
________ Educational apps
________ Reading e-books
________ Watching shows and movies
________ Drawing and creative apps
________ Other (please describe): ____________________________
________ Doesn't use a touchscreen

4. How often does your child use a touchscreen device to learn at home?

________ Several times a day
________ Once a day
________ Several times a week
________ Once a week
________ Less than once a week
________ Never

5. How often does your child use touchscreen devices like tablets/smartphones at school?

________ Several times a day
________ Once a day
________ Several times a week
________ Once a week
________ Less than once a week
________ Never

6. How much do you think your child learns from using touchscreens?

________ A lot

________ Somewhat
________ A little
________ Not at all

7. How often does your child read books or is read to by others at home?

________ Several times a day
________ Once a day
________ Several times a week
________ Once a week
________ Less than once a week
________ Never

8. How often does your child use a book to learn at home?

________ Several times a day
________ Once a day
________ Several times a week
________ Once a week
________ Less than once a week
________ Never

9. How often does your child read books or is read to by others at school?

________ Several times a day
________ Once a day
________ Several times a week
________ Once a week
________ Less than once a week
________ Never

10. How much do you think your child learns from reading books or being read to by others?

________ A lot
________ Somewhat
________ A little
________ Not at all

11. Does your child ever use <u>touchscreen devices</u> for any of the following activities? Check all that apply below.

________ To find information about nature
________ For cooking or looking up recipes
________ To find out the weather report
________ To find directions or geographical information
________ To find information about electrical appliances

________ To find information about sports

12. Does your child ever see you or others use <u>touchscreen devices</u> for any of the following activities? Check all that apply below.

    ________ To find information about nature
    ________ For cooking or looking up recipes
    ________ To find out the weather report
    ________ To find directions or geographical information
    ________ To find information about electrical appliances
    ________ To find information about sports

13. Does your child ever use <u>books</u> for any of the following activities? Check all that apply below.

    ________ To find information about nature
    ________ For cooking or looking up recipes
    ________ To find out the weather report
    ________ To find directions or geographical information
    ________ To find information about electrical appliances
    ________ To find information about sports

14. Does your child ever see you or others use <u>books</u> for any of the following activities? Check all that apply below.

    ________ To find information about nature
    ________ For cooking or looking up recipes
    ________ To find out the weather report
    ________ To find directions or geographical information
    ________ To find information about electrical appliances
    ________ To find information about sports
